# Application, Advancement, and Complication of Ureteral Stent and Encrustation: A Major Complication

**DOI:** 10.7759/cureus.28639

**Published:** 2022-08-31

**Authors:** Mridul Bhardwaj, Nishikant Ingole

**Affiliations:** 1 Medical Education, Jawaharlal Nehru Medical College, Datta Meghe Institute of Medical Sciences, Wardha, IND; 2 Pharmacology, Jawaharlal Nehru Medical College, Datta Meghe Institute of Medical Sciences, Wardha, IND

**Keywords:** complication, application, advancement, encrustation, ureteral stent

## Abstract

Three technological aspects have a significant impact on the functioning of an optimal stent. The substance it is made up of, model or design, and coating of the surface are important areas for research. To give recognition of an ideal stent, it summarizes some essential breakthroughs that occurred. Encrustation is a regular problem that can happen when a ureteral stent is implanted in the urinary tract, and it may be dangerous. The part of the paper covers the mechanism of encrustation, stent management, and the most recent technologies created to solve this problem. Encrustation has a complicated and diverse mechanism that includes the time it stays inside, patient-specific risk factors, controlled film production, formation of biofilm, and deposition of minerals. A number of high-tech advancements in stent substances and coverings/coatings could help to reduce the danger of encrustation of stents. It's critical to determine the amount of encrustation of a stent so that therapy options can be tailored properly. For the care of ureteral stents, which are encrusted, we offer a unique therapeutic protocol. The duration of stent indwelling time has been repeatedly established to be a critical risk factor for the evolution of encrustation. The period of stent indwelling time has consistently been established to be a critical risk element for the evolution of encrustation. Patients who are predisposed to bacteriuria and urinary lithiasis are also predisposed to encrustation. Repeated urinary tract infections, diabetes, and chronic kidney failure are among the factors that might escalate urine bacterial load, which can lead to stent encrustation. Due to the prevalence of ureteral stents in urology, it's critical to keep up to date on the best ways to prevent stent encrustation, recognize high-risk patients, and remove them using multimodal techniques.

## Introduction and background

Ureteral stents were routinely utilized as a solution for short-term or long-term drainage of the obstructed upper urinary tract (UT) over the last few decades [1]. The fundamental reasoning is to let the urine flow to circumvent inner or outer impediments that prevent it from draining properly. Nonetheless, stents have minimal side effects that make their usage and administration more difficult, especially when used as an extended therapeutic choice [2]. The colonization of bacteria and encrustation on the surface of stents are two of the most usual origins of infections which are stent-related and blockage, which can lead to the device's inability to function. Given that these difficulties have such a remarkable impact on therapeutic outcomes, patient well-being of life, and healthcare provider costs, more efforts should be made to effectively address them [2-3].

## Review

Historical perspective

According to a study, ureteral stents have been used since ancient Egypt. Gustav Simon carried out the first ureteral catheterization procedure in 1900, which required inserting a tube into the urine system by surgery, which was an open-bladder one [1]. Joaquin Albarrano invented the earliest ureteral stent with a similar structure to the ones presently in use in the initial 20^th^ century. Subsequently, advances in technology have had a considerable effect on stent blueprint and matter qualities. Each of these innovations was intended to face a particular failing or source of despair. They are reviewed in this paper, along with the concerns they intended to resolve [2-3].

Constitutive materials

Engineers and scientists have spent the last few years determining the best materials for ureteral stents, paying particular attention to tensile strength, pliability, biocompatibility, surface coarseness, and cost-effectiveness [2]. Polymers and metals are the two primary categories of biocompatible materials that are typically used to create ureteral stents [2].

The first ureteral stents were made of the synthetic material polyethylene. However, its use in the therapeutic setting was constrained by rigidity and a propensity to shatter. In order to get around these drawbacks, Gorman et al. developed a polyethylene and polyurethane blend that is more resistant to encrustation [3]. Gort et al. introduced metallic stents. They were designed to lessen stent-related morbidity and strengthen their resistance to deformation brought on by extrinsic or intrinsic ureteral blockage.

Metallic stents, in particular the resonant metallic stent designed by Cook Medical, Indiana, experienced encrustation, as seen upon withdrawal, in an in vivo trial on 50 patients by Liatsikos and others in the year 2009, and as a result, did not produce a substantial depletion in encrustation rates [2-3].

Stent design

The most prevalent form of stent design, first presented by Finney in 1978, is known as the "Double-J." The 'J' form of the stent on each end, which is meant to hold the stent in place and prevent movement or displacement [2]. Finney introduced grooved stents in 1981, which had exterior grooves running the length of the stent lumen. This blueprint was created primarily as therapy after lithotripsy, recourse to help with stone clearing by providing various urine drainage channels [3].

Anderson et al. were the first to introduce spiral stents in the late 1980s. This design had a wire made of metal inside the stent to keep it in a coil form, and it was thought to aid urine outflow in the issue of external occlusion by establishing a steady and long-lasting ureteral lumen aperture. By utilizing the meshed construction to lessen the chance of clogging, self-enlarging meshed ureteral stents were designed with the goal of reducing UT (urinary tract) discomfort and enhancing the flow of urine inside the stent. The classic double-J stent is extremely similar to tail stents [3]. The key structural difference is at the stent's far end, where polymer loops replace the traditional pigtail. The goal of this blueprint was to reduce the irritation in the bladder produced by traditional stents [3].

The construction of double-durometer stents is very much like that of stents which are of tail type. The key distinction is in the stent body's mechanical qualities, which go from firmer at the near termination (kidney) to softer at the far termination (bladder) [3]. Macaluso et al. first introduced the magnetic-tipped stent in 1989, with the goal of reducing the additional expenditures associated with stent removal [2-3]. Other varieties of ureteral stents have been created as a result of modifications to the stent designs already covered. The so-called dual lumen stent is one illustration; it contains two drainage channels to offer compensation if the stent becomes obstructed. Jason Hafron et al. investigated this design in an ex vivo kidney model and found that it resulted in better urine drainage over time than single lumen stents [3]. The resonant metallic stent is another, more recent type that Cook Medical produced, intending to make indwelling stents endure up to one year or longer. It is made of a compressed spring without side holes. Although this stent has a higher initial cost than polymeric stents, the ultimate cost-effective impact is not as substantial because polymeric stents frequently need to be removed and replaced. Additionally, preliminary research done by Wah and others on 15 patients over a year revealed enhanced urine drainage compared to conventional double-J ureteral stents [3].

Complications and side effects

The insertion of a ureteral stent might produce localized inflammation, resulting in haematuria and bladder pain. Reflux of urine toward the kidney might produce discomfort if bladder pressure is elevated. These unfavorable side effects may cause abdominal pain. The placement of a stent might potentially result in stone retrogressive propulsion toward the organ and kidney. It was also shown that stents (ureteral) have a direct impact on ureteral peristalsis, which affects the flow of urine and pressure of the kidney, causing individuals to be more uncomfortable during urination. Renal pelvis inflammation has also been linked to decreased ureteric peristalsis. Stenting has been linked to bladder irritation, which can induce urine urgency and other symptoms [4].

Another big issue is stent migration from its original location. However, from the advent of 'J ends', which have a securing impact and limit movement of the stent over a while, its occurrence has been greatly reduced. Furthermore, ureteral stents made of polyurethane have superior shape memory (and hence efficiently observe the urinary tract) than silicone, minimizing the risk of ureteral stent relocation. Stents made of softer materials, on the other hand, have been proven to be more prone to migration [3]. Stent malposition, relocation, irritative bladder symptoms, pain in lumbar regions, blood in urine and UTI (urinary tract infections), encrustation, and fragmentation of the stent were all examples of ureteral stent problems.

Because ureteral stents are unfamiliar to the urinary system, germs may colonize them and perhaps build biofilms on their surfaces. The ureteral stent may need to be prematurely removed or replaced as a result of this problem. For instance, after two weeks following stent installation, bacterial colonization happened in almost half of 52 patients in research by Pack et al. utilizing Percuflex stents. Another issue that could prevent ureteral stents from remaining in place is encrustation [2]. It happens in conjunction with the existence of bacteria that are known to produce urease, including Proteus mirabilis. These bacteria elevate the pH of urine, which causes crystals to develop. Several variables, including urine composition and pH, stent material and surface qualities, stent dwell time, and urine flow dynamics, could influence stent encrustation. The parts that follow go into additional detail about these issues [4].

Encrustation

The build-up of mineral crystals onto the exterior and cavity of a ureteral stent is known as encrustation. This can cause major issues, particularly with persistent residing stents or unremembered/retained stents, probability in up to 13% of patients. Encrusted stents get ossified (calcified) and fragile, losing their durability and increasing the chances of fracture of the stent or avulsion of the ureter during withdrawal. Deposition of crystal in the stent lumen can block drainage and interact with the ureter's urothelial lining, causing ureteral trauma. Furthermore, prolonged ureteral stent retention was linked to a higher chance of chronic renal disease and hospitalization for UTI (urinary tract infection) or septicemia following the removal of the stent [3-5].

Mechanism

Encrustation is a complicated and varied mechanism. After the stents are implanted, they are immediately layered with a conditioning film which is constituted of glycoproteins appropriate to the individual's body tissues and urine content, which can result in any of three outcomes: 1) the stent might be unchanged, 2) the stent could be progressively covered with a bacterial biofilm (causing urosepsis in the affected individual), or 3) the stent might acquire encrustation [5,6]. Mineral deposition on the surface of the stent always causes encrustation. Encrustation can happen on its own due to high quantities of mineral substances in the urine (oxalate, calcium, phosphorus), or it can be catalyzed by the existence of organisms that are urease-producing, which is very much like how kidney stones develop. Bacteria that are Urease-producing (*Klebsiella*, *Pseudomonas*, *Proteus*, and others) split urea into ammonia, raising the urine pH and causing struvite to form on the stent surface [6].

It is unclear how encrustation and bacterial biofilm development are related. Crystal precipitation and encrustation may be aided by bacterial biofilms. Encrustation, on the other hand, might operate as a hotbed for the growth of bacterial microorganisms and bacterial biofilms, which could lead to urosepsis in affected individuals with pre-fitted stents. However, any substance covering a stent alters its natural physical properties (for example, a protein conditioning layer or bacterial biofilm), which might cause crystals to develop on the stent surface. Additionally, the surface properties of the stent have more time to change and crystallize to form the longer the stent has been in place [7].

Current Stent Technology and Encrustation Prevention

Ureteral stent blueprints and high-tech, additionally their commerce, have evolved substantially. According to the latest study, the worldwide business for ureteral stents was valued at approximately $360 million in 2018, and also it is expected to reach approximately $560 million by 2026 [8]. Because bacterial colonization is a critical factor in the formation of urosepsis and encrustation, past and ongoing analyses have focused on designing matter and/or stent layerings that limit this activity [9]. Nevertheless, bacterial adhesion is a complicated process; this has proven problematic [10]. Organisms have a diversity of mechanisms of adhesion that differ by species/genus; thus, a universal solution is difficult to find [11].

The majority of stents now being used are constituted of polymer mixtures with encrustation-lessening qualities that might or might not be covered with bioactive substances. These mixtures, though frequently proprietary, are typically based on polyurethane. There are other polymer mixtures as well, including polyester and urethane/silicone/PVC hydrogel (Aquavene), styrene/ ethylene-butylene/styrene block copolymers (C-Flex), and others (Silitek). Stents made with proprietary copolymer mixes with polyurethane listed as the major composite material include Silhouette, Bardex, Tecoflex, etc. [12,13].

Diagnosis, Prognosis, and Management

While most stents may be separated without the need for imaging, in individuals with encrustation risk variables, imaging is important to assess and analyze the seriousness and position of encrustation which is along the stent. The degree of encrustation can often be determined using a standard KUB (Kidney Ureter Bladder) X-ray. However, an ultrasound or CT (Computed Tomography) scan may be required to determine the level of encrustation and devise a plan of action for removing a stent that is encrusted [14].

Various grading systems prevail to describe the level of disease and forecast surgical difficulties for stent removal after characterizing the extent of encrustation on imaging [11]. According to the size, position, and grading of encrustation, the Acosta-Miranda et al. FEC ("Forgotten, Encrusted, Calcified") method categorizes encrustation on a grading scale of 1 to 5. The KUB system is another option, which is put out by Arenas et al. It assigns a grade to each section of the stent depending on the degree of encrustation on a scale of 1 to 5 [12-14]. 

Future perspective

Biodegradable stents, which speculatively provide an economic benefit by removing the requirement for another treatment to withdraw the stent and the possibility of stents being forgotten/retained, are now a rich area of research [15]. The continually changing form of the surface of the stent, which could limit the adhesion of bacteria, is another possible advantage in preventing encrustation. In a 2013 in vivo study, Uriprene (Poly-Med Inc., Anderson, SC) was found to have better physiological responses than Polaris (Minnesota, US), although the investigation did not address distinctive rates of encrustation or statistically significant variations in positive urine culture rates.

Researchers are looking into novel ways to stop bacterial adherence and biofilm development to prevent urosepsis and encrustation [16,17]. Some people have looked at stents that were coated with biomaterials such as peptides which were antimicrobial, bacterial-killing enzymes, and essential oils in reaction to worries about resistance offered by bacteria to antibiotic eluting stents. According to a recent study by Hazan et al., Foley urinary catheters connected to lesser energy sound wave producers kept urine sterile for up to a week longer than animals (control) when they were inserted into the male rabbit urethras. Electron microscopy also supported this finding and confirmed the treatment catheters' reduced biofilm burden [2,18,19]. These techniques' therapeutic usefulness hasn't yet been clarified, though. Despite the potential of this research being constrained specified by the cohort population size and illness burden reported, one series of 10 affected individuals with repeated, heavy encrustation loads described no formation of crystalline biofilm in vivo after ureteral stents covered in diamond-like carbon coverings (average indwelling time 97 days) [16,20].

Discussion

Aside from scientific advancements in stent substance, blueprints, and surface layering, the flow active presentation of stents has lately gotten a lot of attention [21]. It's been suggested that the local field of the flow of a stent is linked to the crystal and bacteria deposition. The discoveries have the potential to transform future designs of stents and supplement material and coating advancements [22,23]. While ureteral stents are intended to relieve symptoms and issues associated with a variety of urological disorders, stent encrustation is a significant complication that can have a negative influence on a patient's standard of living and welfare, especially in those individuals who need lasting stenting [20,24-26]. Novel ureteral stent high-tech is a fast-evolving sector that allows physicians, investigators, and medical apparatus makers to interact, which could reduce encrustation and enhance individual results. The present ureteral stent business has a wide range of devices that permit clinicians to customize stent choices to maximize and personalize patient care.

Future studies will focus on characterizing the pathophysiology of the encrustation of stents, improving bio-material properties, and gaining a better understanding of the involvement of the urinary microbiome. The endourologist may face significant difficulties as a result of stent encrustation; hence prolonged indwelling durations should be kept to a minimum. Extracorporeal shock wave lithotripsy and ureteroscopy produce highly favorable results and frequently eliminate the need for more invasive procedures, even though patients frequently need numerous treatments. In some patients, duplex or twin (double) ureteral stenting is a viable alternative to nephrostomy implantation. However, in the latter stages, severe stenosis may necessitate the implantation of a nephrostomy or other more invasive treatments [27-30].

## Conclusions

While the implantation of ureteral stents is intended to relieve the symptoms and consequences of a variety of urological disorders, encrustation of stents is a severe impediment that can negatively affect patient safety and quality of life, especially in those individuals who need prolonged stenting. So as to reduce encrustation and enhance affected individual outcomes, doctors, researchers, and medical device makers can work together in a rapidly. An expanding field of the development of novel ureteral stent technology. The current ureteral stent market provides a range of solutions that enable healthcare professionals to customize the stent choice in order to improve and uniquely manage each patient. This research examines several developments in ureteral stent substances, architecture, and layering. Every one of these advancements is aimed at addressing various causes of stent failure, including encrustation and biofilm formation. Integrating the best substance, blueprint, and layering would enable the creation of an ideal stethoscope. Defining the pathology and physiology of encrustation of stents, enhancing biomaterial properties, and comprehending the involvement of the urinary microbiota will be the focus of future studies. The study of complications of ureteral stents will allow doctors to minimize encrustation and make it a thing of the past.
